# Calcifications valvulaires chez l'hémodialysé au Maroc

**DOI:** 10.11604/pamj.2016.24.115.7147

**Published:** 2016-06-02

**Authors:** Samia Ait Faqih, Béfa Noto-Kadou-Kaza, Lalla Meryam Abouamrane, Naoufal Mtiou, Selma El Khaya, Mohamed Zamd, Ghislaine Medkouri, Mohamed Gharbi Bengahanem, Benyounes Ramdani

**Affiliations:** 1 Service de Néphrologie, de Dialyse et de Transplantation Rénale du CHU Ibn Rochd de Casablanca, Maroc

**Keywords:** Calcification valvulaire, échocardiographie, hémodialysé, prévalence, Valvular calcification, echocardiography, hemodialyzed, prevalence

## Abstract

**Introduction:**

Les calcifications valvulaires constituent une des complications cardiovasculaires majeures de l'hémodialysé de par sa prévalence et son caractère prédictif de morbidité et de mortalité. De nombreux facteurs de risque sont à l'origine de ces calcifications. Le but de notre étude est d’évaluer à la fois la prévalence des calcifications valvulaires chez nos patients hémodialysés ainsi que leurs facteurs de risque.

**Méthodes:**

Il s'agissait d'une étude transversale monocentrique, descriptive et analytique, ayant inclus 111 patients adultes hémodialysés depuis plus de 6 mois au centre d'hémodialyse du CHU Ibn Rochd de Casablanca et qui ont eu à bénéficier d'une ETT durant l'année 2013.

**Résultats:**

L’âge moyen de nos patients était de 44 ± 14 ans. L'ancienneté moyenne en hémodialyse était de 146 ± 80 mois. La pression artérielle moyenne était de 123 ± 23 mmHg pour la systolique et de 72 ± 13 mmHg pour la diastolique, la PTHi moyenne de 529±460 pg/ml, la calcémie moyenne de 86±10 mg/l et la phosphatémie moyenne de 40±15 mg/l. La CRP moyenne était de 11±19,8 mg/L. Sur le plan thérapeutique, 96% des patients étaient sous carbonate de calcium, 11% sous 25 OH vitamine D, 55,5% sous 1 hydroxy-vitamine D3. La prévalence des calcifications valvulaires était de 15% avec une localisation valvulaire aortique dans 41,2% et valvulaire mitrale dans 41,2%. En analyse univariée, seule la durée d'hémodialyse semble être associée à la survenue des calcifications avec p = 0,09 proche du seuil de significativité.

**Conclusion:**

La prévalence des calcifications valvulaires chez nos patients hémodialysés reste élevée même si elle parait relativement moindre comparée aux données de la littérature. Aucun facteur de risque connu n'est apparu significativement associé à ces calcifications.

## Abstract (en)

**Introduction:**

Valvular calcifications are one of the major cardiovascular complications of hemodialysis because of its prevalence and its predictive indices of morbidity and mortality. There are many risk factors associated with these calcifications. Our study aims to evaluate both the prevalence of valvular calcifications in our patients on hemodialysis and their risk factors.

**Methods:**

This was a single-center cross-sectional descriptive and analytical study of 111 adult patients who were on hemodialysis for more than 6 months at the hemodialysis center CHU Ibn Rushd, Casablanca and who underwent ETT during the year 2013.

**Results:**

The average age of our patients was 44 ± 14 years. The average duration of hemodialysis was 146 ± 80 months. Average systolic blood pressure was 123 ± 23 mmHg and average diastolic blood pressure 72 ± 13 mmHg diastolic, average iPTH was 529 ± 460 pg/ml, mean serum calcium was 86 ± 10 mg/l and mean serum phosphate was 40 ± 15 mg/l. Mean CRP level was 11±19,8 mg/L. From the therapeutic point of view, 96% of patients were treated with calcium carbonate, 11% with 25 OH vitamin D, 55,5% with 1 hydroxy-vitamin D3. The prevalence of valvular calcification was 15% with aortic valve location in 41.2% and mitral valve location in 41.2%. In univariate analysis, only hemodialysis duration seems to be associated with the occurrence of calcifications and approaches marginal level of significance (p = 0.09).

**Conclusion:**

The prevalence of valvular calcification in our hemodialysis patients remains high even if it seems relatively low compared to the literature data. No known risk factor was significantly associated with these calcifications.

## Introduction

Les affections cardiovasculaires constituent la première cause de morbidité et de mortalité chez l'hémodialysé. La mortalité est 3 à 20 fois plus élevée par rapport à la population générale, ceci à cause de nombreux facteurs notamment l'accélération du processus d'athérosclérose, d'artériosclérose, d'artériolosclérose et les calcifications vasculaires observées chez les patients urémiques [1]. Parmi ces affections cardiovasculaires, les calcifications valvulaires tiennent une bonne place. En effet il s'agit d'une complication non seulement fréquente, mais aussi constituerait un puissant facteur prédictif de morbidité et de mortalité cardiovasculaire chez l'hémodialysé [2, 3]. De nombreux facteurs de risque à la fois classiques et propres à l’état d'urémie sont à l'origine de ces calcifications [4]. Le but de notreétude est d’évaluer à la fois la prévalence des calcifications valvulaires chez nos patients hémodialysés ainsi que leurs facteurs de risque.

## Méthodes

Il s'agissait d'une étude transversale monocentrique, descriptive et analytique, ayant inclus 111 patients adultes hémodialysés depuis plus de 6 mois au centre d'hémodialyse du CHU Ibn Rochd de Casablanca et qui ont eu à bénéficier d′une ETT durant l′année 2013. Pour chacun de nos patients, nous avions analysé les données démographiques, cliniques, biologiques et échocardiographiques. Les données cliniques ont été recueillies à partir des dossiers des malades et comportent: âge, sexe, durée en hémodialyse en mois, néphropathie initiale, pressions artérielles systolique (PAS) et diastolique (PAD), caractéristiques de l′abord vasculaire, prise de poids inter dialytique (PPID) et dose de dialyse mesurée par Kt/V. Les paramètres biologiques étudiés étaient: calcémie, phosphatémie, cholestérol, HDL-cholestérol, LDL-cholestérol, triglycérides, hémoglobine, ferritinémie, c-réactive protéine (CRP), albuminémie. Pour chacun de ces paramètres, nous avions calculé la moyenne annuelle. Le taux de parathormone intacte (PTHi) retenu était celui effectué au moment de l′étude. L′ETT a été effectuée entre deux séances d′hémodialyse et avait permis de rechercher les calcifications valvulaires au niveau des valves cardiaques. Pour les paramètres de prise en charge des troubles minéralo-osseux, nous avions retenu pour chaque patient la moyenne des doses quotidiennes de carbonate de calcium en grammes par jour et la moyenne des doses hebdomadaires de 1hydroxy-vitamine D3 administrées après chaque séance de dialyse. L′analyse statistique a été réalisée à l'aide du logiciel Sphinx. Les variables quantitatives ont été exprimées en moyenne ± écart-type et les variables qualitatives en pourcentage. Les tests t de Student et le chi 2 ont été utilisés pour la comparaison des variables quantitatives et qualitatives respectivement. Une valeur p < 0,05 est considérée significative.

## Résultats

L’âge moyen de nos patients étaitde 44 ± 14 ans (extrêmes: 18 à 90 ans) avec une prédominance féminine dans 51,4%. L'ancienneté moyenne en hémodialyse étaitde 146 ± 80 mois soit environ 12 ans. La glomérulonéphrite chronique prédominait les néphropathies causales. La pression artérielle moyenne était de 123 ± 23 mmHg pour la systolique et de 72 ± 13 mmHg pour la diastolique. L'hémoglobinémie moyenne était de 10±1,8 g/dL, la PTHimoyenne de 529±460 pg/ml, la calcémie moyenne de 86±10 mg/l et laphosphatémie moyenne de 40±15 mg/l. Le KT/V moyen était de 1,75±0,44. La CRP moyenne était de 11±19,8 mg/L, l'albuminémie moyenne de 43±4,8 g/L et le cholestérol total moyen de 1±0,36 g/L. Sur le plan thérapeutique, 96% des patients étaient sous carbonate de calcium à la dose moyenne de 2 g/j, 11% sous 25 OH vitamine D, 55,5% sous 1 hydroxy-vitamine D3 à la dose moyenne de 1,74µg/semaine (Tableau 1). Les calcifications valvulaires ont été notées chez 17 patients soit une prévalence de 15% avec une localisation valvulaire aortique dans 41,2% et valvulaire mitrale dans 41,2% également (Tableau 2). En analyse univariée, seule la durée d'hémodialyse semble être associée à la survenue des calcifications avec p = 0,09 proche du seuil de significativité Tableau 3, Tableau 4.

**Tableau 1:** Données épidémiologiques des hémodialysés

Données	Hémodialysés (n = 111)
Données cliniques	
Age (moyenne±DS), année	44 ± 14
Sexe féminin, n (%)	57 (51)
Données d'hémodialyse	
Durée dialyse (moyenne±DS), mois	146 ± 80
Néphropathie initiale, n(%)	
Diabète	7(6,2)
Glomérulonéphrite chronique	19(17,1)
Néphropathie indéterminée	50(45,0)
Néphroangiosclérose	6(5,4)
Autres	29(26,1)

**Table 2:** Données cliniques et biologiques des hémodialysés

Données	Hémodialysés (n = 111)
Données cliniques	
IMC(Kg/m2)	
PAS (mmHg)	123±23
PAD (mmHg)	72±13
PAM (mmHg)	90± 22
Données biologiques	
Hb(mg/dl)	10±1,8
Alb(g/l)	43±4,8
Ca (g/l)	86±10
Ph(g/l)	40±15
PTHi(pg/l)	529±460
CT(g/l)	1±0,36
KT/V	1,75±0,44
CRP(mg/l)	11±19,8

IMC: Index de masse corporel PAS: Pression artérielle systolique PAD: Pression artérielle diastolique PAM: Pression artérielle moyenne Hb: hémoglobine Alb: albumine CA: calcium PH: phosphate CT: cholestérol total

**Tableau 3:** Données sur les calcifications valvulaires

Données	Hémodialysés (n = 17)
Localisation, n(%)	
Valvule Aortique	7(41,2)
Valvule Mitrale	7(41,2)
Valvule mitroaortique	3(16)

**Tableau 4:** Facteurs de risque des calcifications valvulaires en analyse univariée

Données	Calcifications		P
	Oui = 17	Non = 94	
**Age (moyenne±DS), année**	48	44	0,31
**Sexe(%)**			
Masculin	52,9	48,9	0,83
Féminin	47,1	51,1	
**Ancienneté en dialyse (moyenne±DS), mois**	146	141	0,09
PTHi(pg/l)	495	535	0,73
CT(g/l)	1	1,06	0,51
Ca (g/l)	86,65	86,15	0,83
Ph(g/l)	44	40	0,22
CaCo3(g/j)	2,06	2,02	0,85
1OHVitD3 (ug/sem)	1,53	1,78	0,72
PAM (mmHg)	94	90	0,48

PAM: Pressionartériellemoyenne Ca: calcium Ph: phosphate CT: cholestérol total, PTHi: parathormoneintacte CaCo3: carbonate de calcium 1OHVitD3: 1 hydroxyvitamineD3 active

## Discussion

Les calcifications valvulaires chez l'insuffisant rénal chronique en général, et chez l'hémodialysé en particulier constituent une complication majeur en terme de prévalence et de risque de mortalité. Il s'agit d'une affection fréquente dont la prévalence peut atteindre jusqu’à 50% [2]. Dans notre étude, elle est de 15%. Cette prévalence est similaire à celles de Ezziani et al. [5], et Ellouali et al. [6] au Maroc qui ont rapporté respectivement des prévalences de 14% et 22,6% dans leurs études. Par contre elle est plus faible par rapport à celle rapportée par Mihailescue et al. en Roumanie qui est de 57 > % [7]. La faible prévalence de calcifications valvulaires dans notre étude comparée à celle de Mihailescue et al. Pourrait être due en partie à l’âge jeune de nos patients, 44 ans contre 57 ans; à la moindre sévérité des troubles minéralo-osseux dans notre étude, le PTHi moyen de nos patients étant de 529 ug/l, comparé au 1113 ug/l rapporté par Mihailescue et al. Ces calcifications touchent de façon variable les différentes valves. Dans notre, la prévalence des calcifications aortiques et mitrales sont identiques. Mihailescue et al. ont rapporté 57% de calcifications mitrales et 40% de calcifications aortiques. La prévalence élevée de calcifications valvulaires chez l'hémodialysé est due à de nombreux facteurs de risque [3]. Il existe deux types de facteurs à savoir les facteurs cardiovasculaires classiques tels que l’âge avancé, le diabète, la dyslipidémie; et ceux propres à l'urémie elle-même et à la dialyse tels que les troubles minéralo-osseux en particulier l'hyperparathyroidie secondaire, la supplémentation en sels de calcium, le syndrome inflammatoire, la durée d'hémodialyse [4]. Dans notre étude aucun facteur de risque n’était associé de façon significative à la survenue de calcifications valvulaires. Les mécanismes physiopathologiques sous-jacents des calcifications valvulaires restent à démontrer. Certains auteurs suggèrent un processus actif plutôt qu'une précipitation minérale passive. PourMohler et al [8], il s'agirait d'un même processus que celui conduisant à la calcification de plaque athérosclérotique. Ce processus implique caractérisé les monocytes, les macrophages et les cellulesosteoblastlike et, les proteines caractéristiques de la matrice osseuse telles que matrice proteine GLA, les enzymes telles que la métalloprotéinase [9–23]. La présence des calcifications valvulaires est un puissant facteur prédictif de toute cause de mortalité et de mortalité cardiovasculaire chez ces patients. Ce risque est d'autant plus élevé que le nombre de valves calcifiées augmente témoignant de l’étendue des calcifications [24]. La progression de des calcifications est supérieure àcelle observée chez les patients âgés avec une fonction rénale normale. Dans une étude, la progression annuelle moyenne du degré de sténose aortique est de0,23 cm2/an contre 0,05 à 0,1 cm2/an chez les patients non urémiques [25]. Il est important de dépister ces calcifications souvent silencieuses afin d'instaurer des stratégies thérapeutiques pouvant prévenir ou retarder les calcifications vasculaires et valvulaires en corrigeant essentiellement les désordres du métabolisme phosphocalcique. Du fait de l’évolution rapide du rétrécissement aortique chez l'hémodialysé, la présence de calcifications aortiques justifie un contrôle échographique deux fois par an afin de prévenir une décompensation cardiaque [26].

## Conclusion

La prévalence des calcifications valvulaires chez nos patients hémodialysés reste élevée même si elle parait relativement moindre comparée aux données de la littérature. Aucun facteur de risque connu n'est apparu significativement associé à ces calcifications. Cependant étant une cause connue de morbimortalité, seule une surveillance régulière à la recherche des complications permettra d'instaurer une thérapeutique adéquate.

### Etat des connaissance sur le sujet

- Les calcifications valvulaires chez l'hémodialysé constituent un facteur majeur de risque cardiovasculaire;
- Les calcifications valvulaires chez l'hémodialysé constituent un facteur prédictif de morbidité et de mortalité.

### Contribution de notre étude à la connaissance

- Notre étude confirme que la prévalence reste élevée aussi bien dans les pays développés que dans les pays en voie de développement;
- En dehors des facteurs de risque décrit par la littérature, la durée moyenne d'hémodialyse pourrait également constituer un autre facteur de risque.
